# Effects of Low Versus Moderate Glycemic Index Diets on Aerobic Capacity in Endurance Runners: Three-Week Randomized Controlled Crossover Trial

**DOI:** 10.3390/nu10030370

**Published:** 2018-03-17

**Authors:** Krzysztof Durkalec-Michalski, Emilia E. Zawieja, Bogna E. Zawieja, Dominika Jurkowska, Maciej S. Buchowski, Jan Jeszka

**Affiliations:** 1 Institute of Human Nutrition and Dietetics, Poznan University of Life Sciences, 60-624 Poznań, Poland; emilia.zawieja@gmail.com (E.E.Z.); dominika.jurkowskadj@gmail.com (D.J.); jeszkaj@up.poznan.pl (J.J.); 2 Department of Mathematical and Statistical Methods, Poznań University of Life Sciences, 60-637 Poznań, Poland; bogna13@up.poznan.pl; 3 Energy Balance Laboratory, Department of Medicine, Vanderbilt University, Nashville, TN 37232, USA; maciej.buchowski@vanderbilt.edu

**Keywords:** glycemic index, nutrition, aerobic capacity, endurance, running performance, body composition

## Abstract

The glycemic index (GI) of ingested carbohydrates may influence substrate oxidation during exercise and athletic performance. Therefore, the aim of this study was to assess the effect of low- and moderate-GI three-week diets on aerobic capacity and endurance performance in runners. We conducted a randomized crossover feeding study of matched diets differing only in GI (low vs. moderate) in 21 endurance-trained runners. Each participant consumed both, low- (LGI) and moderate-GI (MGI) high-carbohydrate (~60%) and nutrient-balanced diets for three weeks each. At the beginning and end of each diet, participants had their aerobic capacity and body composition measured and performed a 12-min running test. After LGI, time to exhaustion during incremental cycling test (ICT) and distance covered in the 12-min run were significantly increased. The MGI diet led to an increase in maximal oxygen uptake ($\dot{V}$O_2_max), but no performance benefits were found after the MGI diet. The LGI and MGI diets improved time and workload at gas exchange threshold (GET) during ICT. The results indicate that a three-week high-carbohydrate LGI diet resulted in a small but significant improvement in athletic performance in endurance runners. Observed increase in $\dot{V}$O_2_max on MGI diet did not affect performance.

## 1. Introduction

Carbohydrates and fat are the main sources of fuel oxidized in muscles during endurance exercise [1]. In contrast to fat, endogenous stores of carbohydrates are limited. Exercise-induced hypoglycemia contributes to the perception of fatigue and consequently attenuates athletic performance [2]. However, while the ingestion of adequate amounts of carbohydrates is vital to maintain glucose availability and optimize athletic performance [1], the carbohydrate quality may also play an important role because various carbohydrate-rich foods affect postprandial glycaemia differently.

The glycemic index (GI) classifies carbohydrate-containing foods based on their postprandial glycemic response [3]. The GI of a food is defined as the incremental area under the two-hour blood glucose response curve (AUC) following consumption of the tested food usually containing 50 g of CHO and divided by the AUC of a reference food containing the same amount of CHO (either glucose or white bread) and multiplied by 100. In general, foods with a high GI increase blood glucose concentration more rapidly than foods with a low GI. The GI of food is termed low when it is below 55, medium when it is between 55–70, and high when it is above 70 [4,5,6]. Initially, the GI was used to design meals and diets for patients with diabetes, but current applications include weight loss and improvement in athletic performance [7,8,9,10,11].

The effects of a pre-exercise meal’s GI on aerobic capacity and endurance performance have been investigated with equivocal results. Some studies reported improved endurance capacity or performance after ingestion of low-GI (LGI) compared to a high-GI (HGI) meal before exercise [9,10,12,13], while others have not [7,8,14]. These discrepancies could be caused by tested meal’s carbohydrate content and GI, meal timing, study design, and type of exercise test. For example, in two studies [8,9] with similar exercise protocols, cyclists were provided with a pre-exercise meal containing 1 g∙kg^−1^ CHO and ingested 45 min before with meals, but the obtained results were different. In a study by Kern and colleagues [8], no differences in performance on the 15-min cycling trial were found, while in the study by Moore and colleagues [9], performance on the 40-km time trial significantly improved. This discrepancy might have been caused by differences in the GIs between the test meals (moderate vs. high and low vs. high) or by different performance cycling tests used in the Kern at al [8] and Moore et al. [9] studies (15 min vs. >90 min), respectively. Nevertheless, one meta-analysis concluded there is no clear benefit of low-GI pre-exercise meal for endurance performance regardless of carbohydrate ingestion during exercise [15], while another meta-analysis found that endurance performance following an LGI meal is superior to that following an HGI meal [16].

The available literature on the effect of diets with high versus low GI fed for 3–5 days on endurance performance or exercise capacity is limited to a few studies with inconclusive results. For example, Chen et al. [17] showed that the most important factor in improving athletic performance was ingesting a three-day high-carbohydrate diet regardless of diet’s GI. Hamzah et al. [18] found that consuming a five-day high-carbohydrate diet with either high or low GI had no impact on time to exhaustion or distance covered during a treadmill test at 65% maximal oxygen uptake ($\dot{V}$O_2_max). However, it is possible that three- or five-day diet might be too short to elicit any metabolic changes and alter athletic performance. The intake of carbohydrates in these studies was relatively high (>70%), potentially limiting changes in substrate oxidation and restricting possible beneficial metabolic adaptations. It is possible that long-term low-GI diets might provide greater metabolic alterations than short-term diets. A plausible physiological rationale for long-term LGI diets is an increased fat oxidation caused by reduced carbohydrate availability during exercise. In addition, increased availability of non-esterified fatty acids could enhance the mitochondrial enzymes activity [19]. Therefore, training on a LGI diet can be a good strategy for improving endurance adaptations, but more research in this area is necessary. We hypothesized that in actively training athletes a high-carbohydrate diet with low GI compared with moderate GI consumed for three weeks would induce modest improvements in aerobic capacity (maximal oxygen uptake, gas exchange threshold). Our secondary hypotheses were that endurance performance (distance in the 12-min running test and time to exhaustion) and maximal workload in the incremental cycling test (ICT) would differ between low and moderate GI diets for three weeks. To test these hypotheses, we conducted a randomized crossover feeding trial in a group of young actively training endurance runners consuming diets with either low (LGI = 39 ± 1) or moderate (MGI = 69 ± 1) GI for three weeks.

## 2. Materials and Methods 

### 2.1. Participants

Eligibility criteria were age 17 years or older, self-reported good health, current medical exam confirming person’s ability to practice sports, at least three years of endurance running training, and currently training four or more times/week for 1.5–2 h/day. We included both males and females because of equal participation of both genders in endurance running.

Exclusion criteria included current smoking or illicit drug use, alcohol consumption greater than 1–2 drinks/week, and dietary supplements use or being on any special diet less than three weeks before the study. For females, additional exclusion criteria were being pregnant or planning to become pregnant during the study.

The primary recruitment strategy was contacting potential participants through the local runners’ clubs using mailings, flyers, and word-of-mouth. The recruitment goal was 20 participants completing two diet periods and all exercise tests. After initial telephone screening, 36 potential participants (13 females) were invited to the study. At the screening visit, 11 participants were found ineligible or not interested in the study. Twenty-five runners (9 females), either professional or recreational long- (*n* = 14) and middle-distance (*n* = 11) runners, were enrolled into the study. Each running subgroup (professional and recreational, long and middle distance) followed the same training schedule for the entire study (~10 weeks).

**Figure 1:**
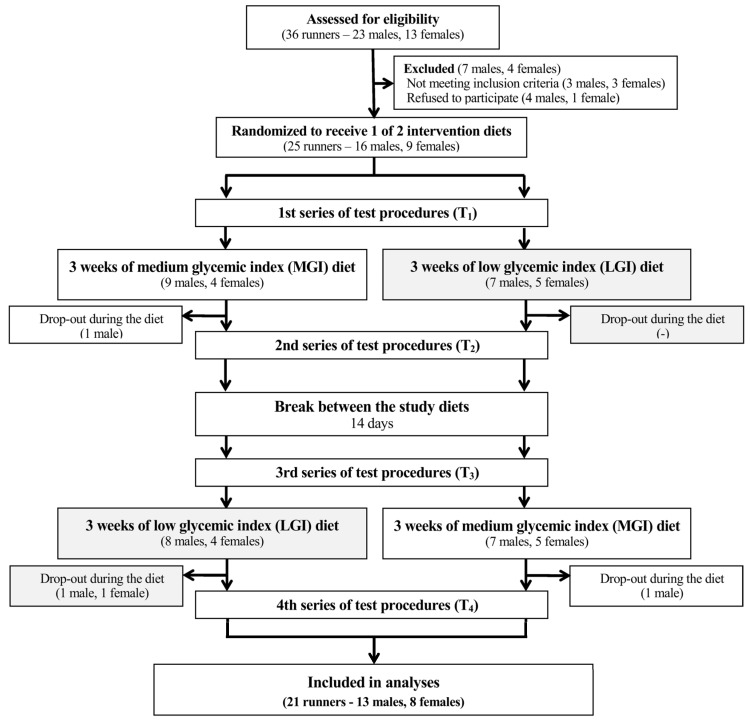
A flow chart of the study design. Abbreviations: GI—glycemic index, MGI—moderate glycemic index, LGI—low glycemic index.

The study protocol was reviewed and approved by the local institutional review board (Bioethics Committee at Poznań University of Medical Sciences, reference number: 173/15 of 5 February 2015). In accordance with the Declaration of Helsinki, all participants and parents of participants younger than 18-year-old signed informed consents. The trial was conducted from February to April 2015. This trial was registered at Clinical Trials Gov (website: https://clinicaltrials.gov/show/NCT03062527; Clinical Trial Identification Number: NCT03062527). The study was registered retrospectively since the registration was not required when the study enrolment started. The authors confirm that all ongoing and related trials for this intervention are registered. The study complies with the Consolidated Standards of Reporting Trials (CONSORT) Statement for randomized trials as shown in Figure 1 and Table S1.

### 2.2. Study Design and Protocol

Enrolled participants were randomized to the group receiving MGI or to LGI diet (based on their lean body mass). The random allocation sequence was performed using stratified randomization by impartial biostatistics. All participants began a 14-day run-in phase during which they ate their habitual diet and recorded all ingested food and beverages in food diaries (Table 1). Collected intake data were analyzed and used to determine general eating habits and assess each individual’s diet compatibility with the study diets. During this period, total daily energy expenditure was estimated using a wrist-worn heart rate monitor (Polar RS-400, Polar, Vantaa, Finland) worn for four consecutive days, as described previously [20]. After the run-in phase and the first laboratory study visit followed by an endurance test on the next day, participants started dietary intervention. In a crossover design, participants consumed in a random order MGI or LGI diets for three weeks each and separated by a two-week break (Figure 1). The diet length (three weeks) was chosen based on the study by Clapp and Lopez [21] in adult women showing that 20 days of low-GI diet caused significant metabolic alterations. The break between diets (two weeks) was chosen based on previous studies showing that 10–14 days is sufficient time to prevent carryover of potential metabolic changes [18,22]. Participants were instructed to maintain the same physical activity level throughout the study.

#### 2.2.1. Study Visits

The protocol included four visits to the laboratory before and after the first (T_1_ and T_2_) and the second (T_3_ and T_4_) diet. At each visit, body mass and composition were measured and followed by the exercise tests and the field endurance test on the next day. On the following day, participants began their prescribed (either LGI or MGI) three-week diet ending with the T_2_ visit. Three hours before T_2_ and T_4_ visits, participants consumed a standardized MGI or LGI meal corresponding to a second meal on the first day from the received menus (Tables S2 and S3). The three-hour period between the meal and exercise was chosen to prevent potential short effect of a meal on exercise performance [12,23,24]. The LGI meal (GI = 39) consisted of wholegrain rye bread, dried tomatoes, low-fat quark, cucumber, lettuce and dried apricots. The MGI (GI = 65) meal consisted of low-fat quark, wheat bread, dried tomatoes, dried raisins, and plain yoghurt. During the two-week break between the study diets, participants followed their self-selected diet. After the break, participants had their T_3_ visit and began the second (MGI or LGI) three-week diet culminating in the T_4_ visit.

#### 2.2.2. Study Diets

MGI and LGI diets were individualized to ensure weight maintenance throughout the dietary interventions. Diets provided 11–15% of energy from protein (1.6 g∙kg body mass^−1^), 25% energy from fat, and 60–64% from carbohydrate as recommended by the International Society of Sports Nutrition [25] (Table 1). Diets were developed considering the type and GI of foods, cooking methods, and pre-established macronutrient ratios. The GI was calculated from the published tables [4,5,6]. Carbohydrate-containing foods in the LGI diet included wholegrain rye bread, rolled oats, oat bran, brown rice, buckwheat, wholegrain pasta, vegetables except for corn and potatoes, and fruits such as apples, grapefruits, tangerines, dried apricots, and unripe bananas. Carbohydrate products in the LGI diet were cooked *al dente* to keep the GI as low as possible. The major sources of carbohydrate in the MGI diet were wheat bread and pasta, potatoes, instant oats, cornflakes, white rice, vegetables, and fruits such as ripe bananas, grapes, raisins, dates, and cranberries, honey, and high-sugar jams. Carbohydrate products in MGI diet were slightly overcooked to increase the GI. Daily diets were divided into 5–6 moderate- size meals to prevent digestive problems caused by consumption of large food portions.

The participants received seven–day menus (Tables S2 and S3) for the LGI or MGI three-week diet. All instructions about foods and meal preparation were provided by the study dieticians. The participants were encouraged to contact the dieticians with any questions or concerns about the diets.

For food intake recording, the participants used food diaries and electronic kitchen scales. Study dieticians gave instructions on how to use the scales and complete the diaries to each participant individually. Participants recorded time and the amount of foods and beverages consumed at each meal, the amount of leftovers, and any deviations from the diet.

The diaries were collected at T_2_ and T_4_ visits. A dietician reviewed and discussed the diary with each participant. The energy and nutrient intake from diaries were calculated using Dietetyk-2 software (JuMar 2006, Poznań, Poland).

#### 2.2.3. Anthropometry and Body Composition

Body mass and height were measured in duplicate using a calibrated scale with a stadiometer (WPT 60/150 OW, Radwag^®^, Radom, Poland) in a fasted state, to the nearest 0.1 kg and 0.1 cm. Fat mass and fat-free mass were assessed by air displacement plethysmography (Bod Pod^®^, Cosmed, Rome, Italy) [26]. Total body water content was assessed using bioelectric impedance (BIA 101S, Akern-RJL, Pontassieve, Italy) [27].

#### 2.2.4. Daily Energy Expenditure

Total daily energy expenditure was assessed using heart rate (HR) monitoring data (Polar RS-400, Vantaa, Finland), based on a previously validated method [20]. Each participant’s HR was recorded minute-by-minute for four consecutive days. To eliminate any accidental errors (e.g., cell phone interference, loss of skin contact during sleep) in HR recordings, participants were asked to report time and type of habitual daily activities and training. The information was used to fill the potential gaps in HR recordings. Recorded wrist-worn HR data were downloaded to a computer equipped with the Polar ProTrainer 5 program (ver. 5.41.002, Vantaa, Finland). On a separate visit, the thresholds HR (HR_FLEX_) for activity categories (sedentary, light, moderate and vigorous) were estimated individually for each participant. Energy expenditure (EE) for each category was calculated as recommended, and recorded four-day HR data were categorized to the intensity levels and used to estimate total daily energy expenditure [20,28].

#### 2.2.5. Exercise Tests

The exercise tests were conducted at four visits (T_1__–__4_) and included aerobic capacity and endurance running performance tests. Although all participants were familiar with the tests from previous studies and training, they were encouraged to ask questions about the details of the protocol.

##### Aerobic Capacity (ICT Test)

The incremental cycling test (ICT) was conducted under standardized conditions (temp. 20–22 °C and humidity 60–70%) between 08:00 and 10:00. The aerobic capacity was assessed from the maximal oxygen uptake ($\dot{V}$O_2_max) and gas exchange threshold (GET). Following ~5 min warm-up on cycloergometer (Kettler-X1, Kettler, Ense-Parsit, Germany), a participant started the incremental test at the workload of 50 W for females and 75 W for males at 70 ± 5 rpm. Every 1.5 min, the workload increased by 25 W until reaching maximum perceived exhaustion assessed using the Borg scale (1–20) [29,30]. The respiration indices were recorded using a calibrated ergospirometer (Quark CPET, Cosmed, Rome, Italy) and analyzed using Cosmed CPET software (ver.9.1b, 2010). $\dot{V}$O_2_max was identified when a workload increase stopped generating a further increase in oxygen uptake (V_O_2__) and HR [29,30]. GET was determined using the V-slope method [31]. The ICT was chosen to eliminate factors inherent to treadmill testing such as different running technique and type of shoes that could cause measurement errors [32].

##### Endurance Performance

Endurance performance was defined as the distance covered (meters) during the 12-min running test [33] conducted in an open-field athletic stadium in small groups (4–6 runners) between 14:30 and 18:00 (temp. 8–11 °C and humidity 54–66%). The 12–min running test was chosen since it applies to both to middle and long-distance runners participating in our study.

#### 2.2.6. Statistical Analysis

The study was powered to detect a difference in maximum oxygen uptake (based on our pilot study in this population) between the two diets. A sample of individuals will achieve 90% power to detect a 0.115 L∙min^−1^ difference in maximum oxygen uptake, assuming alpha = 0.05.

The results are presented as means ± standard deviation (and 95% confidence intervals). Data were analyzed using a two-way repeated measures ANOVA with the inclusion of experimental diets order (MGI first or LGI first). This analysis allowed the elimination of carryover effect. Post hoc analysis was done using Bonferroni correction. When the sphericity assumption was violated, the Greenhouse-Geisser and the Huynh-Feldt corrections were performed. To compare the intake of energy, macronutrients, and dietary fiber, as well as the GI of study diets, Student’s *t*-test or Wilcoxon test was performed, depending on the normal or not normal data distribution, respectively. Statistical significance was set at *p* < 0.05 and data were analyzed using the STATISTICA-12 software program (StatSoft Inc., Tulsa, OK, USA).

## 3. Results

### 3.1. Participants and Adherence

From 25 randomized participants, four did not complete the study either due to injury (one female and two males) or not adhering to the protocol (one male). Twenty-one participants (eight females) completed both dietary periods and four visits (T_1–4_) and reported no significant lifestyle and training routine changes during the study. The compliance with prescribed diet was high (99–104% for energy and different nutrients). Intakes of energy, macronutrients, dietary fiber, and GI did not differ between prescribed diets and data from dietary records (all: *p* > 0.05) (data not presented).

### 3.2. Body Mass and Composition

There were no significant differences between MGI and LGI diets at the beginning and the end of each trial (Table 2). Body mass and body composition did not change after the MGI diet, but after the LGI diet, body mass was 0.8 kg lower than after MGI diet (*p* = 0.0483) (Table 2).

### 3.3. Aerobic Capacity

$\dot{V}$O_2_max increased significantly after the MGI diet compared to baseline (*p* = 0.0159), whereas time to exhaustion improved after the LGI diet compared to baseline (*p* = 0.0474) (Table 3). However, after adjusting for body mass, no changes in $\dot{V}$O_2_max were observed. The order of diet (first MGI or first LGI) was significant for time to exhaustion, time to GET, and workload at GET, but it was excluded thanks to adequate statistical analysis. Maximum workload did not change after both diets (Table 3). HR_max_ differed significantly after both LGI and MGI diets (Δ = +3 bpm for LGI (*p* = 0.0001) and Δ = −3 bpm for MGI (*p* = 0.0359)). Significant differences were also found in HR_max_ before MGI and LGI diets (*p* = 0.0002) and after MGI and LGI diets (*p* = 0.0163). Time to GET increased significantly by ~0.6 min after MGI compared to baseline (*p* = 0.0091) and ~0.5 min after LGI diet (*p* = 0.0003) (Table 3). workload at GET (W_GET_) increased significantly after MGI diet (*p* = 0.0459) and after LGI diet (*p* < 0.0001) compared to baseline. Furthermore, HR_GET_ did not change throughout the study.

### 3.4. Endurance Performance

**Figure 2:**
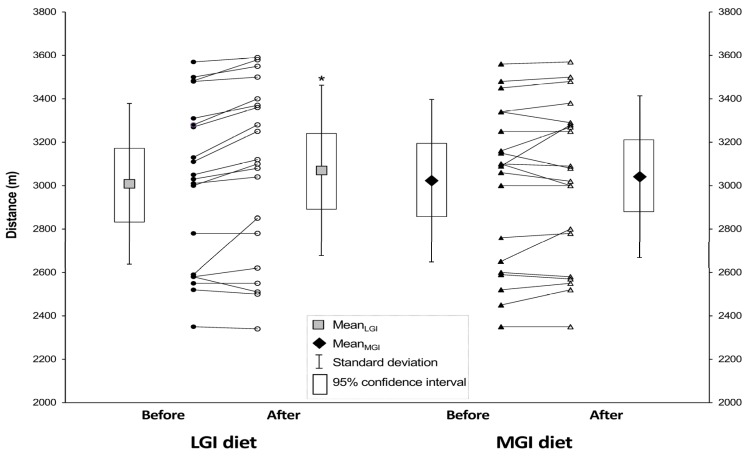
Mean distance covered in the running test before and after the three-week LGI and MGI diets. Values are means ± SD (and 95% confidence intervals). Abbreviations: MGI—moderate glycemic index, LGI—low glycemic index. * Significantly different from baseline (*p* = 0.0015).

The mean distance covered on the 12-min running test was significantly (*p* = 0.0015) longer after LGI diet (3070 ± 393 m) compared to baseline (3008 ± 370 m) but did not differ before and after MGI diet (Figure 2). Moreover, the order of the diets (first LGI or first MGI) was also significant (*p* = 0.0085) for the results in 12-min running test, but this effect was excluded in analysis thanks to proper statistical method.

## 4. Discussion

To the best of our knowledge, this is the first study to examine the effect of diets differing in GI over a relatively long time (three weeks). The major findings are that the LGI diet consumed for three weeks by actively training endurance runners may improve distance covered in the 12-min running test and time to exhaustion in ICT. Gas exchange threshold was improved by both experimental diets. Although we observed statistically significant differences in HR_max_ within diets, we do not consider these differences clinically important [34]. The LGI diet caused a slight decrease of body mass. However, our findings have to be interpreted with caution and need to be confirmed in larger trials conducted in various athletes’ populations.

The effect of GI on athletic performance was investigated previously, but the results were inconsistent. Several studies assessed the effect of a single pre-exercise meal with a different GI on endurance performance and found that either the performance was improved after low-GI meal or GI had no influence on performance [9,10,12,35,36]. In contrast, other studies showed that a pre-exercise low-GI compared to high-GI meal improved running capacity by 7–23% [13,37]. Several studies assessed the effect of a longer, usually 3–5 day, diets with various GI on athletic performance. For example, in a study of Hamzah and colleagues [18], after five days of either high-GI or low-GI diet, no differences were found in running capacity (time to exhaustion—high GI: 107 min vs. low GI: 110 min) [18]. Similarly, Jamurtas et al. [7] did not find a difference in time to exhaustion after the pre-exercise low-GI and high-GI meals. We were not able to find any previous reports that used GI-controlled diet for longer than one week, making an interpretation of our results difficult. Specifically, we found that compared to baseline, the 3-week LGI but not the MGI diet improved time to exhaustion in ICT and distance covered in the 12-min running test. Also, the differences between LGI and MGI diets were not significant. In addition to longer diet time, there were other methodological differences between our and other studies [17,18]. We measured performance using distance covered in the 12-min running test and time to exhaustion and maximal workload in the ICT, both of which demand high intensity effort. The ICT used in the present study to assess aerobic capacity, was chosen to eliminate factors inherent to treadmill test such as different running technique and type of shoes that could cause measurement errors [30]. The previous short-term studies [12,13,24,38,39] used lower intensity performance tests, and since at higher workloads fat oxidation is downregulated and CHO utilization increases [40], our results cannot be compared to the results in these reports.

In this study, distance covered in the 12–min run significantly increased after LGI diet, indicating a possible improvement in performance endurance. It has been reported that a low-GI meal ingested before an endurance exercise resulted in a stable blood glucose concentration during the test [12,24,37,38,41]. In contrast, a high-GI meal caused a rapid decrease in blood glucose concentration after 10 to 20 min of exercise, despite the fact that postprandial glucose concentration was higher after high-GI meal [35,37,40]. Lower glucose concentration after high-GI meal was also observed at the end of exercise [12,24]. That might have been caused by higher insulinemic response to the high-GI meal than the low-GI meal and a resultant glucose clearance from bloodstream. In our study, the tests were conducted 2–3 h after a meal at all visits and lasted for approximately 12–13 min (running test: 12 min; incremental cycling test: from 12.5 to 13.5 min on average). Since the drop in glucose concentration following high-GI meal from 10 to 20 min after the onset of exercise was previously reported [35,37,38], we surmised that the longer distance covered after the LGI than the MGI diet might have been caused by the differences in blood glucose level, glycogen storage, and usage between the diets and/or better adaptation to more stable glycaemia. However, the interpretation is limited since glucose availability was not measured in the present study.

Although exercise performance is ultimately the most important end-goal for an athlete, monitoring aerobic capacity (GET, $\dot{V}$O_2_max) is important for determining the impact of training and nutritional strategies on the athlete’s capabilities. In the present study, baseline values of $\dot{V}$O_2_max were slightly lower before the MGI than the LGI diet, but they were similar post-MGI and post-LGI diets. In addition, $\dot{V}$O_2_max adjusted for body mass was not different between the diets.

Both diets increased time to GET and W_GET_, suggesting an improved aerobic metabolism. It was reported previously that the anaerobic threshold is correlated with maximal fat oxidation [42] and the point where fat utilization becomes negligible [43]. Therefore, the occurrence of GET later and at higher exercise intensities may suggest better fat oxidation capacity. However, those assumptions are rather intuitive due to the lack of detailed metabolic data. In previous studies, fat oxidation was generally enhanced after the consumption of LGI meal compared to HGI meal [12,13,24].

From the endurance athletes’ point of view, achieving and maintaining low body and fat mass is beneficial since it might improve performance [44,45,46]. In the present study, the LGI diet induced a 0.6 kg body mass decrease. Body mass was significantly lower after LGI compared to after MGI diet. In a study by Hamzah et al. [18], a five-day low-GI diet did not induce changes in body or fat mass. The difference between the studies might be caused by the diet duration (three weeks vs. five days). More research on the effects of a long-term LGI diet in combination with energy restriction on body weight and composition in athletes is warranted. Such studies could be especially important in sports that use a rapid weight reduction before competition such as wrestling [47,48]. Although low-GI diets have been shown to promote weight loss and improve lipid profiles in obese and overweight adults [11], little research has been reported on the influence of the GI on body composition in endurance athletes. Our study adds to the literature by highlighting the possibility that a relatively long (e.g., three weeks) LGI diet might help endurance athletes to accomplish modest changes in body weight and composition.

The limitations include small sample size and self-reported adherence to the diets. Although participants reported high adherence and diet compliance, it is possible that some did not eat all foods or ate foods they did not report. Four participants did not finish the study. However, considering the protocol requirements, 16% attrition seems reasonable. Relatively small sample size did not allow us to assess the effect of gender and age differences on study outcomes. An additional limitation was that we used the 12-min running test for assessing the potential effect of diets on aerobic capacity. However, the test is well known and commonly performed by runners and thus does not require familiarization and allows for evaluation running performance. In addition, we also used the incremental cycling test, which measures aerobic capacity more accurately than the 12-min running test. Finally, we did not measure blood biochemical parameters during and after exercise, making it impossible to identify the mechanism of GI effect on exercise performance and endurance.

The strengths include a relatively homogeneous population of actively-trained endurance runners, a comparatively long three-week dietary intervention, and using reference standard methods to assess differences between the diets in a randomized crossover design.

Future research should focus on confirming our results in a larger and various populations and assessing the difference in aerobic capacity and performance changes between diets with high and low GI. Practical application of our results is that athletes and coaches might consider the GI of food in planning a healthy diet, as the LGI diet might slightly improve performance and help to achieve desirable body composition.

## 5. Conclusions

Compared to baseline, the LGI diet consumed for three weeks resulted in a small but significant improvement time to exhaustion and running performance in endurance-trained runners. Gas exchange threshold was improved by both diets. Our results have to be interpreted with caution, and more research is needed to understand better the effect of long-duration diets with a various GI on body mass and composition, aerobic capacity, performance, and possibly metabolic adaptation to exercise.

## Figures and Tables

**Table 1:** Composition of the baseline, moderate glycemic index (MGI), and low glycemic index (LGI) diets.

Parameter	Baseline MGI_before_	MGI Diet	Baseline LGI_before_	LGI Diet
Energy intake (kcal)	3060 ± 632	3190 ± 616	3062 ± 651	3174 ± 600
Protein (g)	141.2 ± 33.1	109.0 ± 20.8 *	141.6 ± 33.8	109.7 ± 20.6 ^‡^
Fat (g)	102.8 ± 20.4	90.4 ± 16.2 *	103.5 ± 21.2	90.4 ± 17.5 ^†^
Carbohydrates (g)	392.5 ± 83.4	485.2 ± 101.3 *	389.8 ± 86.8	480.2 ± 96.8 ^‡^
Dietary fiber (g)	31.7 ± 2.6	38.1 ± 6.5 ^#^	31.9 ± 2.4	57.6 ± 8.0 ^‡§^
Glycemic Index (GI)	60 ± 3	63 ± 1 ^#^	60 ± 3	39 ± 1 ^‡§^

Values are means ± standard deviation (SD). The differences between diets were analyzed using Student’s *t*-test or Wilcoxon test depending on the distribution of data (normal—not-normal). Significantly different from MGI before: * *p* < 0.0001, ^#^ *p* = 0.0001; Significantly different from LGI before: ^‡^ *p* < 0.0001, ^†^ *p* = 0.0001; Significantly different from MGI diet: ^§^ *p* < 0.0001.

**Table 2:** Body mass and body composition before and after the three-week MGI and LGI diets.

Parameter		ANOVA	MGI Diet	LGI Diet	Carryover Effect
		*p*-Value			*p*-Value
Body mass (kg)	Before	0.0311	65.3 ± 11.3	65.7 ± 11.9	0.1153
			(60.2–70.5)	(60.2–71.1)	
	After		65.9 ± 12.0	65.1 ± 11.7 ^#^	
			(60.4–71.3)	(59.7–70.4)	
Fat-free mass (kg)	Before	0.5954	51.2 ± 8.4	51.3 ± 9.0	0.2541
			(47.4–55.0)	(47.3–55.4)	
	After		51.4 ± 8.4	51.2 ± 8.6	
			(47.6–55.2)	(47.2–55.1)	
Fat mass (kg)	Before	0.3828	14.1 ± 4.3	14.3 ± 4.7	0.3064
			(12.2–16.1)	(12.2–16.5)	
	After		14.5 ± 5.0	13.9 ± 4.5	
			(12.2–16.8)	(11.8–16.0)	
Total body water (L)	Before	0.2268	37.6 ± 6.2	38.0 ± 6.5	0.5317
			(34.8–40.4)	(35.0–41.0)	
	After		37.7 ± 6.2	37.8 ± 6.3	
			(34.9–40.5)	(34.9–40.7)	
Fat-free mass (%) ^$^	Before	0.8168	78.5 ± 4.3	78.4 ± 4.8	0.4444
			(76.6–80.5)	(76.2–80.6)	
	After		78.3 ± 4.8	78.9 ± 4.4	
			(76.1–80.5)	(76.9–80.9)	
Fat mass (%) ^$^	Before	0.8168	21.5 ± 4.3	21.6 ± 4.8	0.4444
			(19.5–23.4)	(19.4–23.8)	
	After		21.7 ± 4.8	21.1 ± 4.4	
			(19.5–23.9)	(19.1–23.1)	
Total body water (%) ^$^	Before	0.6500	57.7 ± 4.8	58.1 ± 4.3	0.6636
			(55.6–59.9)	(56.1–60.0)	
	After		57.6 ± 5.1	58.3 ± 4.0	
			(55.3–59.9)	(56.5–60.1)	

Values are means ± SD (and 95% confidence intervals). Abbreviations: MGI—moderate glycemic index, LGI—low glycemic index. ^#^ Statistically different form after MGI (*p* = 0.0483). ^$^ percent of body mass (kg).

**Table 3:** Aerobic capacity indices before and after the three-week MGI and LGI diets.

Parameter		ANOVA	MGI Diet	LGI Diet	Carryover Effect
		*p*-Value			*p*-Value
$\dot{V}$O_2_max (L∙min^−1^)	Before	0.0397	3.39 ± 0.86	3.45 ± 0.87	0.5905
			(3.00–3.78)	(3.05–3.85)	
	After		3.52 ± 0.84 *	3.51 ± 0.82	
			(3.14–3.90)	(3.14–3.88)	
$\dot{V}$O_2_max (mL∙min^−1^·kg^−1^)	Before	0.1130	51.58 ± 9.43	52.26 ± 8.52	0.2673
			(47.29–55.87)	(48.38–56.14)	
	After		53.38 ± 8.76	53.90 ± 8.70	
			(49.39–57.37)	(49.94–57.86)	
Texh (min)	Before	0.0174	12.6 ± 3.5	12.6 ± 3.2	0.0318
			(11.0–14.2)	(11.1–14.1)	
	After		13.0 ± 3.4	13.3 ± 3.3 ^§^	
			(11.4–14.6)	(11.8–14.8)	
Wmax (W)	Before	0.1559	267 ± 72	267 ± 66	0.0951
			(234–299)	(237–297)	
	After		268 ± 66	276 ± 68	
			(238–298)	(245–307)	
HRmax (bpm)	Before	0.0000	182 ± 8 ^‡‡^	179 ± 8	0.2626
			(178–186)	(175–183)	
	After		180 ± 9 ^#^	182 ± 9 ^II §§^	
			(176–184)	(178–186)	
T_GET_ (min)	Before	0.0001	10.5 ± 2.5	10.6 ± 2.6	0.0097
			(9.4–11.7)	(9.4–11.8)	
	After		11.1 ± 2.8 ^‡^	11.1 ± 2.6 ^¶^	
			(9.8–12.4)	(9.9–12.3)	
HR_GET_ (bpm)	Before	0.0785	171 ± 10	169 ± 10	0.2203
			(166–175)	(165–174)	
	After		172 ± 9	171 ± 9	
			(168–176)	(167–175)	
W_GET_ (W)	Before	0.0000	226 ± 53	226 ± 52	0.0043
			(205–250)	(202–250)	
	After		236 ± 57 ^†^	240 ± 50 ^††^	
			(210–262)	(217–263)	

Values are means ± SD (and 95% confidence intervals). Abbreviations: MGI—moderate glycemic index, LGI—low glycemic index, $\dot{V}$O_2_max—maximal oxygen uptake, T_exh_—time to exhaustion, W_max_—maximal workload, HR_max_—maximal heart rate, GET—gas exchange threshold, T_GET_—time to GET, HR_GET_—heart rate at GET, W_GET_—workload at GET. Significantly different from before: * *p* = 0.0159, ^#^ *p* = 0.0359, ^‡^ *p* = 0.0091, ^†^ *p* = 0.0459, ^§^ *p* = 0.0474, ^II^ *p* = 0.0001, ^¶^ *p* = 0.0003, ^††^ *p* < 0.0001; MGI_before_ significantly different from LGI_before_: ^‡‡^ *p* = 0.0003; LGI_after_ significantly different from MGI_after_: ^§§^ *p* = 0.0163.

## Supplementary Materials

The following data are available online at http://www.mdpi.com/2072-6643/10/3/370/s1. Table S1. CONSORT 2010 checklist of information to include when reporting a randomised trial; Table S2. Examples of Low Glycemic Index Diet (GI = 39) menus; Table S3. Examples of Medium Glycemic Index Diet (GI = 63) menus.

## Availability of Data and Material

The datasets used and/or analyzed during the current study are available from the corresponding author on reasonable request.
